# GALENOS approach to triangulating evidence (GATE): transforming the landscape of psychiatric research

**DOI:** 10.1192/bjp.2025.10457

**Published:** 2025-11-07

**Authors:** Katharine A. Smith, James Downs, Emma S. J. Robinson, Gin S. Malhi, Jennifer Potts, Thomy Tonia, Georgia Salanti, Andrea Cipriani

**Affiliations:** Department of Psychiatry, University of Oxford, UK; NIHR Oxford Health Clinical Research Facility, Oxford Health NHS Foundation Trust, Warneford Hospital, Oxford, UK; and Oxford Precision Psychiatry Lab, NIHR Oxford Health Biomedical Research Centre, Oxford, UK; MQ Mental Health Research, London, UK; School of Physiology, Pharmacology & Neuroscience, University of Bristol, UK; Department of Psychiatry, University of Oxford, UK; Academic Department of Psychiatry, Kolling Institute, Northern Clinical School, Faculty of Medicine and Health, The University of Sydney, NSW, Australia; and CADE Clinic and Mood-T, Royal North Shore Hospital, Northern Sydney Local Health District, St Leonards, NSW, Australia; Department of Psychiatry, University of Oxford, UK; and Oxford Precision Psychiatry Lab, NIHR Oxford Health Biomedical Research Centre, Oxford, UK; Institute of Social and Preventive Medicine, University of Bern, Switzerland; Institute of Social and Preventive Medicine, University of Bern, Switzerland; Department of Psychiatry, University of Oxford, UK; NIHR Oxford Health Clinical Research Facility, Oxford Health NHS Foundation Trust, Warneford Hospital, Oxford, UK; and Oxford Precision Psychiatry Lab, NIHR Oxford Health Biomedical Research Centre, Oxford, UK

**Keywords:** Living systematic review, evidence synthesis, triangulation, risk of bias, co-production

## Abstract

There is an urgent need for better evidence-based interventions in mental health. High-quality randomised controlled trials in humans are often lacking, especially when dealing with complex situations or novel therapeutic targets. Other potentially useful data may be available, such as from early-phase trials, observational or mechanistic studies or animal experiments. Triangulation offers an opportunity to consider a wider variety of evidence together to prioritise future research directions, and ultimately to inform clinical decisions. Here we describe GATE (the GALENOS Approach to Triangulating Evidence). This is the methodology of triangulation, co-produced with people with lived experience, and applied as an integral part of the GALENOS project (Global Alliance for Living Evidence on aNxiety, depressiOn and pSychosis; https://www.galenos.org.uk/). We outline the considerations of triangulation in psychiatry and our experience to date in assessing animal and human data together, using triangulation to prioritise future research directions. With GATE at its core, GALENOS not only enables novel insights to emerge, but points us towards a future of collaborative research better equipped to examine the most pressing questions in mental health.

Mental disorders are an immense cause of distress and impairment worldwide and, as such, contribute significantly to the overall global burden of disease.^1^ Existing research evidence guides our current approaches to treatments, which play an important role in recovery for many people. However, for a significant number of individuals many of the available treatments are insufficient, even when used in combination. Therefore, there is an urgent and growing need for better evidence-based interventions in mental health that focus not only on alleviating symptoms but also on preventing first episodes and relapses. Developing the evidence base for these new interventions – alongside new applications of existing treatments – requires careful and considered prioritisation of the particular mental health research routes that are most likely to yield new and effective therapies, but to date this has been challenging.

## The challenges of accessing evidence to prioritise research in mental health

### Limited RCT evidence

The ‘gold standard’ of evidence when assessing the efficacy of treatments is usually held to be methodologically robust systematic reviews and meta-analyses of high-quality randomised controlled trials (RCTs). However, in psychiatry, RCT evidence can be limited. This is especially true for novel treatments, for specific populations of patients (such as pregnant women, children and adolescents and people with intellectual disability and/or neurodivergent conditions) and for those with a degree of ‘treatment resistance’ when first-line treatments have failed to produce an adequate response. This is further compounded by other factors: research funding is more limited in mental health and, for the most mentally unwell patients, participation in RCTs may pose ethical and practical challenges.^2^ The result is that there are research and clinical questions that have ‘hollow’ systematic reviews, where no evidence can be found that reaches the criteria for inclusion.

### Exponential increase in publications

In contrast to the limited availability of RCTs, there has been an exponential increase in the overall number of scientific publications in mental health. However, this increasing number makes it difficult to appraise and translate research advances in a timely and rigorous way.^3^ Taken together, these challenges can result in decision-making in research and clinical settings that is often left to individual assessment or opinion, rather than being guided by robust evidence.^4^

### Limited high-quality mechanistic research

Mental health interventions are often targeted at symptoms rather than the underlying mechanisms. Both are important, but mechanistic research is essential for the successful development, exploration and application of new and repurposed interventions.^5^ Research in animals can help with mechanistic insights and, while cellular models and experimental medicine approaches in humans provide useful information, human and animal research are both required to investigate the underlying mechanisms of a potential treatment target.^5^ However, each approach has different challenges; for animal research there are challenges associated with animal models limiting interpretation of these data,^6^ and the historical separation of human and animal research has also previously made it more difficult to assess their evidence together.

### Need for co-production with clinicians and people with lived experience

The tension between clinical experience and research evidence in guiding clinical decision-making is longstanding; this is partly because these are often framed as being in opposition. However, while they represent different ways of knowing, clinical practice inevitably involves both – decisions require not only knowledge but also the skill to apply this effectively, and so clinical expertise and involvement in appraising evidence and prioritising research is key.

In addition, while there is an ambition in clinical settings for individuals and their caregivers to be at the centre of shared decision-making, research processes have often excluded people with lived experience and not always focused on the outcomes that matter most to them.^7^ Nevertheless, subjective experiences of illness, treatment and recovery can offer insights that neither research nor clinical expertise alone can fully capture. These insights can challenge assumptions, highlight blind spots and inform more meaningful approaches to care.^8^ Integrating the perspectives of those with lived experience within existing research structures and decision-making practices requires a methodological reorientation in psychiatry, but taking a multidimensional, evidence-based approach to extracting insights from the existing evidence base has proved challenging to date.^9^

### Triangulation as a possible approach to research prioritisation

This Feature explores the potential of triangulation, a methodologically defined but underutilised approach in mental health research. This can be used to assess the available evidence to provide prioritisation for future research, which in turn can then help to inform our understanding of psychiatric conditions, their underlying mechanisms and their management. It is particularly pertinent in psychiatry where, despite great advances in some areas within the field, such as genetics, imaging and pharmacology, we have been unable to find the bedrock of our disorders.

The process of triangulation aims to bring together various sources of evidence while maintaining the highest standards of methodological rigour and expert input. Evidence is much broader than just the pinnacle of the ‘evidence pyramid’ that clinicians and academics are taught in their training. In considering only the RCT evidence, we may be excluding potentially useful and relevant data. All evidence sources are biased in some way, and the different sources of bias can lead to under- or overestimation of the treatment effect in different directions. By drawing on multiple sources of evidence, including for example clinical data from different study designs and preclinical data, and with the voices of people with lived experience included throughout, we can use the power of multiple sources as a way to bridge gaps between research, clinical expertise and lived experience. This will inform research prioritisation, which in turn can provide new evidence to support clinical care.^9^

### What is triangulation and how can it be used in psychiatry and mental health science?

Triangulation is a methodology that has already been used in other fields such as social sciences, education research and epidemiology, and we are now applying it in psychiatry and mental health research.^10–13^ Triangulation refers to the process of collecting, examining, comparing and interpreting evidence from multiple sources to address the same question, ultimately synthesising and integrating the findings.^14^

Triangulation explicitly acknowledges that systematic errors (or biases) are present in each source of evidence, but it assumes that these biases are likely to be unrelated when different study approaches are used. If the results of several different approaches all point to the same conclusion after accounting for the different biases, this will strengthen confidence in the findings.^12^ Triangulation is an important tool because it enables the integration and appraisal of evidence from different sources that would not usually be considered together. For example, the evidence from animal studies is often limited by shortcomings in the animal models available and their translational validity in humans, and by issues of replicability and potential biases.^15^ In addition, clinical data from early-phase studies are sparse and dose–response studies are not easy to conduct. These data from animal studies, mechanistic studies and early-phase clinical studies would not usually be captured by a typical systematic review, and human and animal data are usually considered in separate analyses. Triangulating animal and human evidence from all study designs can therefore be particularly helpful to identify promising research routes and to enable closer examination of the underlying mechanisms that underpin disorders and their treatment.

### GATE

The GALENOS Approach to Triangulating Evidence (GATE) is a structured, multidisciplinary approach to synthesising evidence in mental health, and we hope that this will provide a ‘gateway’ to a more inclusive approach to methodological evidence synthesis in psychiatry and beyond. Triangulation of evidence is the key methodology underpinning GALENOS (Global Alliance for Living Evidence on aNxiety, depressiOn and pSychosis;^9^ https://www.galenos.org.uk/), a Wellcome-funded project that brings together a unique combination of multidisciplinary expertise including people with lived experience. GALENOS focuses on mental illness with priority setting in anxiety, depression and psychosis, and includes both drug and non-drug treatments. It examines diagnostic, prognostic and predictive tools in mental illness where a broad evaluation of the evidence is key, because these areas are rarely examined using RCTs. Expertise comes from different routes: researchers and clinicians with experience in human mental health disorders, experts in non-human (animal) data, experts in evidence synthesis methodology (including triangulation) and people with lived experience. The project works together to generate living systematic reviews (LSRs^16^) in the most challenging and urgent areas of mental health, and uses triangulation to inform the prioritisation of next steps for research, including finding new therapeutic targets, understanding mechanisms (including those for existing drugs) and the role of prognostic factors in modifying response.

#### The steps involved in GATE

**Fig. 1:**
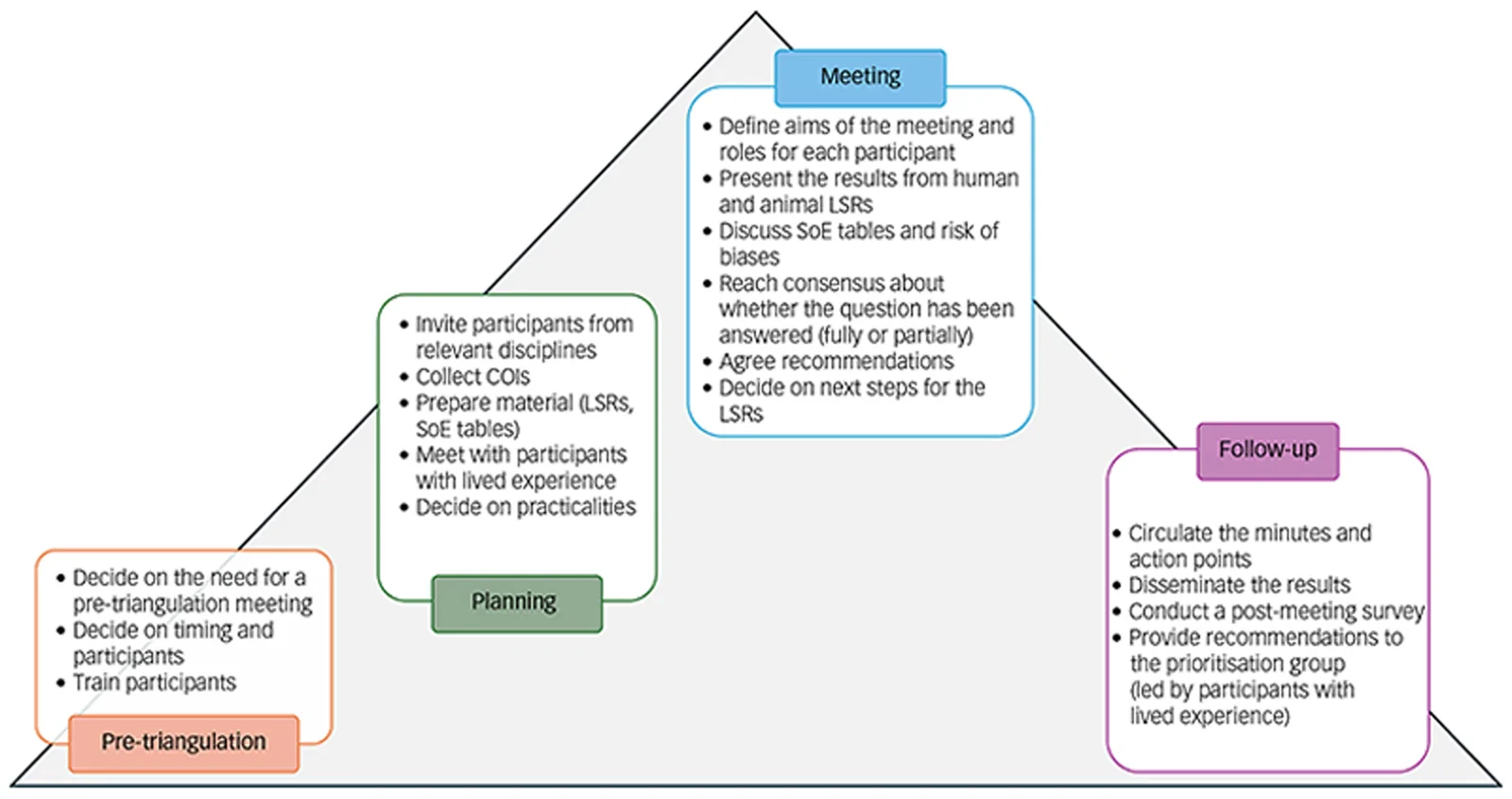
The triangulation process. COIs, conflicts of interest; LSRs, living systematic reviews; SoE, summary of evidence.

This is a new application of triangulation and combines different types of expertise. As a new application, GATE needs to encompass state-of-the-art systematic review approaches while also ensuring the highest standards through iterative training and gathering of multidisciplinary expertise. GATE follows a prespecified sequence. Topics are chosen from the feedback of clinicians, researchers and people with lived experience on priority questions within anxiety, depression and psychosis. After refining a topic of interest, LSRs are produced with published prespecified protocols. The results of the LSRs are then used to inform the triangulation process, implemented in one or more triangulation meetings. GATE describes the collection, evaluation and integration of the diverse types of evidence, including the practicalities of organisation, and conduct of the triangulation meetings (summarised in Fig. 1).

#### Considerations in the processes of GATE (see Fig. 1)

(a) Pre-triangulation
  When gaps, ambiguities or disagreements exist in the LSR protocols – such as uncertainty in definitions, outcomes or study designs – a conceptual refinement phase may be necessary. A pre-triangulation meeting (see Fig. 1) may be needed when LSR protocols require further refinement, especially if there is disagreement over patient/population, Fig. 1 The triangulation process. intervention, comparison and outcomes (PICO) elements, study design selection or if the topic is novel or broad. These meetings help clarify definitions, focus the scope and resolve early methodological conflicts.
(b) Co-production
  The benefits of including people with lived experience alongside academic and clinical experts are best realised when all those involved in co-production are able to be active contributors across the triangulation process. First-hand insights need to be viewed as a relevant and valuable basis to contribute to the interpretation, synthesis or appraisal of evidence across sources, shaping how knowledge is produced and what conclusions are drawn.^17^ A key principle is ‘epistemic pluralism’ – i.e. valuing different ways of knowing alongside one another, including clinical insight, methodological expertise, basic science knowledge and experiential understanding.^18^ This also needs to sit alongside an understanding that all contributions to the process of triangulation may bring an element of bias from each individual. For the lived experience contribution, this can be mitigated to some extent by the inclusion of ‘expert’ contributions and also from inclusive and diverse voices.^19^ In GATE, diverse contributions are involved from a global international group. The process of GATE itself also mitigates these individual biases by including equal contributions from a variety of experts each with different biases and expertise.
  For triangulation to fulfil its potential in mental health science, it must not only diversify evidence sources but also democratise the processes of analysis and decision-making. This involves providing training and support for a diverse range of people with relevant lived experiences to enable them to participate both equitably and ethically, and includes an understanding of the impacts of participation, which may be both positive or negative for the individual or the group. For example, harm or distress may inadvertently be caused by actual or perceived power imbalances, exclusionary language and/or the emotional burden of taking part.^20^ In addition, it is equally important to support the whole research team (including academics and clinicians) to be able to engage with different perspectives and sources of knowledge, and to appreciate how different forms of evidence are legitimised. This requires making space for ongoing dialogue, mutual learning and the co-creation of a shared language for the co-creation of knowledge.^21^
(c) Evaluating the multiple sources of evidence and reaching consensus
  A multidisciplinary and diverse triangulation panel includes clinical and in vivo animal experts, methodologists and people with lived experience, facilitated by one or two co-chairs. The research team presents the LSR reports and summaries of evidence and the panel considers the multiple sources of evidence and their biases. Focused deliberations aim to assess agreement between data sources, account for biases and interpret the findings. GATE encourages a non-binary view of the conclusions of each evidence thesis. Rather than seeking definitive answers, conclusions are considered along a spectrum, allowing for uncertainty and evolving interpretations. This nuanced approach enables recommendations that are flexible and responsive to new data. The triangulation output may range from clear conclusions to refined questions or calls for more data. GATE is not merely about reaching consensus, but also about understanding where and why evidence converges or diverges, and what that means for action or further inquiry.
(d) Deciding about next steps
  Reflection and iteration are integral to GATE, and triangulation is part of an evolving learning process. Feedback, open dialogue and transparent reporting are critical to refining both the methodology and the questions being asked. In essence, GATE is a process of collaborative, integrative knowledge-making grounded in inclusivity, critical thinking and respect for complexity.^22^

### What have we learned from GATE and the individual GALENOS projects so far?

#### Assessing animal and human data together

A unique feature of GATE is the opportunity to consider animal and human data together. An example of this is in the assessment of trace amine-associated receptor 1 (TAAR1) agonists in psychosis.^23^ In this case, the animal data (using a model of locomotor activity induced by a pro-psychotic drug treatment) suggested a potential antipsychotic effect, whereas the human data were more nuanced, showing a lower efficacy for TAAR1 agonists than existing antipsychotic drugs but a more favourable side-effect profile. GATE enabled an assessment of these data and provided recommendations for priorities for future research, and for future iterations of the LSRs.

Even though we did not carry out a formal cost-effectiveness analysis, this is also an example of how GATE allows the effective cross-fertilisation of ideas and approaches across areas that have been increasingly siloed. This approach has also been useful in highlighting the limitations associated with widely used animal models (see ‘Novel insights’, below), and the need for investment in the development of methods for both animal and human research that can improve integration of research findings.

GATE also allows the triangulation of information from healthy controls and patient population data. For example, in an assessment of cognitive bias modification for social anxiety, trials are extracted that involve two different populations: those with a diagnosis of social anxiety and those participants who have been subjected to a social stressor, both being compared with healthy controls.^24^ This allows the synthesis of evidence from a clinical patient population with that of an experimental manipulation that mimics the possible underlying processes of the disorder.

#### Prioritising future research directions

GATE has been used to prioritise future research directions, as in the recent consideration of pro-dopaminergic drugs for anhedonia in depression. Triangulation of the data from animal and human LSRs suggested that pro-dopaminergic interventions reduced anhedonia, but that this may act on an indirect pathway that probably involves other neurotransmitters and needs further mechanistic research.^25^ Not only does this prioritise future research, but it may have clinical implications once more data have been gathered.

#### Training and expertise

Training and gaining expertise in methodology are essential elements in ensuring that triangulation is performed systematically. Just as in a systematic review, the process should be transparent, with clearly defined steps. In GATE these have followed several core processes – for example: (a) the LSR production team presents the results to the triangulation team and answers any factual questions, but importantly they are not involved in the decision-making part of the triangulation process itself; (b) the process is kept on track by independent co-chairs from different backgrounds – for instance, in recent iterations these have been two co-chairs with expertise in methodology and lived experience leadership, respectively.

#### Novel insights

As well as an emphasis on rigorous methodology, the process of the triangulation itself has enabled additional insights. Gathering together experts from different fields has facilitated discussion and highlighted broader concepts that need further research. For example, the first triangulation meeting on TAAR1 agonists in psychosis also discussed the wider challenges in animal research and its translation to clinical psychiatry.^26,27^ Selection bias, performance bias, detection bias and attrition bias are all more common issues in animal studies than in human studies, and reproducibility of findings in animal experiments has proved to be challenging.^15^ There has been progress in this area and there are now several tools to support researchers in planning and reporting studies, as well as risk-of-bias tools for assessing animal studies, but publication bias and selective reporting continue to be present in the animal literature.^6^ New, open research practices, including the pre-registration of preclinical animal studies will help, but systemic change will also be needed within the research community.^15,28^

In terms of measuring clinical symptoms, the triangulation meeting of the evidence on anhedonia and pro-dopaminergic drugs in depression also identified the wider difficulties of measuring anhedonia effectively, consideration of whether it is a separate symptom or an integral part of clinical depression and the lived experience of its key role in impaired function during depression.^29^ Triangulation can also be very useful in systematically exploring concepts that have previously been difficult to define and research. For example, in a recent topic within GALENOS on circadian disruption and its relationship to mood disorders, the challenges of designing the protocol required a ‘pre-triangulation’ meeting (see Fig. 1) to define the parameters and concepts to be studied.

### Where do we go from here?

#### Refining the methodology of GATE

GATE is a systematic process, and its principles and context have been outlined here. Next steps include the production of a detailed methodology summary and a summary of the co-production principles we are following. These will serve as guidance documents not only for the GALENOS project, but also as templates for other researchers. These will be updated as more projects are completed.

#### Co-production

Central to GATE and GALENOS is a commitment to co-production that goes beyond tokenistic or predetermined approaches to involving people with lived experience in research. We recognise that meaningful co-production requires more than inclusion – it also demands resourcing, recognition and reciprocal support.^30^ This has meant offering remuneration to people with lived experience: for example, for GATE we followed the guidance provided by the National Institute for Health and Care Research (NIHR),^31^ supporting flexible and accessible ways of participation and respecting the emotional labour that is often involved.^32^

Building on this foundation, our next steps include systematic evaluation of our co-production processes to understand their impacts, both on evidence synthesis and on strengthening relationships across disciplines and knowledge systems. We are developing frameworks to assess how lived experience contributions enhance transdisciplinary working, with the aim of generating transferable models for meaningful and equitable co-production in complex global health challenges. For example, we have identified a need for more rigorous tools to match the specificity and relevance of lived experience to the research question at hand, recognising that not all experiences are interchangeable, and that the value of lived experience lies not only in having experienced something personally but in being able to reflect on and relate it meaningfully to the focus of inquiry.

#### Automation and artificial intelligence

The GALENOS group has already started to use artificial intelligence methodology to streamline some of the processes of data extraction used in the LSRs. While this is not part of GATE itself, the LSR data are central to the consideration of the evidence within triangulation. For example, automation is already being used for reference selection, with automated data extraction planned in the near future. The process is also supported by the ‘GALENOS crowd’, who will contribute to evidence synthesis activities. The model of crowd-sourcing builds on previous and ongoing successful examples, such as screening and data extraction by the Cochrane crowd^33^ and the MHCOVID crowd.^34^ Members receive training on systematic review methodology and the specific systematic review on which they are working. They receive certificates of training, and their contributions are recognised by acknowledgement or co-authorship in the publications. This also increases opportunities globally for people to access training and increase expertise. This model of automation, combined with crowd-sourcing, ensures ongoing and rigorous updates across multiple LSRs.

#### Global collaborations

Consideration of evidence should not be constrained behind paywalls or restricted only to higher-income countries. GALENOS also focuses on global collaborations at all levels: in data extraction, within GATE and in the subsequent prioritisation process. In total, the GALENOS team has members from 35 countries bringing a diverse range of perspectives and expertise, with recent additions of research fellows from Nigeria, South Africa and Uganda. In addition, an international advisory board of experts and stake-holders aims to ensure that the findings are relevant globally and across high-, middle- and low-income contexts.

#### Open access

Open access to all is essential; GALENOS is not only focusing on producing LSRs and triangulation but also contributing to the wider clinical, research and methodology community. All protocols and up-to-date LSRs are made openly available through Wellcome Open Research (https://wellcomeopenresearch.org/galenos). Curated and harmonised data-sets are made available via a purpose-built repository according to the findability, accessibility, interoperability and reusability (FAIR) principles via the GALENOS data repository (https://www.galenos.org.uk/). Findings will also be categorised using a methodology developed specifically by GALENOS for an open access ontology.^35^

#### Future directions

The need for a greater understanding of the existing evidence on risk factors and mechanisms of action is evident, to inform future research and, ultimately, new treatments that matter to patients.^36^ We champion research programmes that fulfil these goals. However, these projects are expensive and take time, with the lead time to new medications sitting at more than a decade, and so efficient use of resources, including the growing interest in repurposing existing molecules, is key. GALENOS endeavours to do this by harnessing the knowledge that we already have and making the most of it by extracting as much evidence from it as is possible. GALENOS – and GATE as a central part of it – make this possible and are already providing novel insights as well as pointing us to the next steps for future research.

## Data Availability

Data availability is not applicable to this article as no new data were created or analysed in this study.
